# A life course approach to explore the biological embedding of socioeconomic position and social mobility through circulating inflammatory markers

**DOI:** 10.1038/srep25170

**Published:** 2016-04-27

**Authors:** Raphaële Castagné, Cyrille Delpierre, Michelle Kelly-Irving, Gianluca Campanella, Florence Guida, Vittorio Krogh, Domenico Palli, Salvatore Panico, Carlotta Sacerdote, Rosario Tumino, Soterios Kyrtopoulos, Fatemeh Saberi Hosnijeh, Thierry Lang, Roel Vermeulen, Paolo Vineis, Silvia Stringhini, Marc Chadeau-Hyam

**Affiliations:** 1 Department of Epidemiology and Biostatistics, School of Public Health, Imperial College London, London, UK; 2 INSERM, UMR1027, Université Toulouse III-Paul Sabatier, 31000 Toulouse, France; 3 Fondazione IRCCS- Instituto Nazionale dei Tumori, Milan, Italy; 4 Istituto per lo Studio e la Prevenzione Oncologica (ISPO Toscana), Florence, Italy; 5 Department of Clinical Medicine and Surgery, University of Naples Federico II, Naples, Italy; 6 Piedmont Reference Centre for Epidemiology and Cancer Prevention (CPO Piemonte), Turin, Italy; 7 Cancer registry and Histopathology Unit, Azienda Ospedaliera ‘Civile –M.P.Arezzo’, Ragusa, Italy; 8 National Hellenic Research Foundation, Institute of Biology, Pharmaceutical Chemistry and Biotechnology, Athens, Greece; 9 Institute for Risk Assessment, Division of Environmental Epidemiology, Utrecht University, Utrecht, the Netherlands; 10 HuGeF, Human Genetics Foundation, Torino, Italy; 11 MRC-PHE Centre for Environment and Health, Imperial College, London, London, UK; 12 Institute of Social and Preventive Medicine, Lausanne University Hospital, Lausanne, Switzerland

## Abstract

Lower socioeconomic position (SEP) has consistently been associated with poorer health. To explore potential biological embedding and the consequences of SEP experiences from early life to adulthood, we investigate how SEP indicators at different points across the life course may be related to a combination of 28 inflammation markers. Using blood-derived inflammation profiles measured by a multiplex array in 268 participants from the Italian component of the European Prospective Investigation into Cancer and Nutrition cohort, we evaluate the association between early life, young adulthood and later adulthood SEP with each inflammatory markers separately, or by combining them into an inflammatory score. We identified an increased inflammatory burden in participants whose father had a manual occupation, through increased plasma levels of CSF3 (G-CSF; *β* = 0.29; P = 0.002), and an increased inflammatory score (*β* = 1.96; P = 0.029). Social mobility was subsequently modelled by the interaction between father’s occupation and the highest household occupation, revealing a significant difference between “stable Non-manual” profiles over the life course versus “Manual to Non-manual” profiles (*β* = 2.38, P = 0.023). Low SEP in childhood is associated with modest increase in adult inflammatory burden; however, the analysis of social mobility suggests a stronger effect of an upward social mobility over the life course.

It has now been established that health discrepancies exist across socioeconomic groups worldwide^1^. In particular, socioeconomic disadvantages over the life course has been related to poor health in adulthood^2^,^3^. Several epidemiological studies have shown that health behaviors and lifestyle factors are important drivers of social inequalities but cannot fully explain the socioeconomic gradient in health^4^,^5^. One suspected mechanism linking socioeconomic position (SEP) and health outcomes over time involves a differential activation of a wide range of physiological and biological reactions^6^. One important biological mechanism used to adapt to the environment is the stress response systems^7^ controlling the release of stress hormones, whose levels alter many biological mechanisms including inflammatory and immune responses^8^.

Inflammation is a key pathway involved in the development of chronic diseases including cardio-metabolic disorders and multiple cancers^9^. Several studies have examined the influence of SEP on inflammatory marker levels, and showed that lower education andor income was associated with a greater burden of inflammation^10^,^11^,^12^,^13^,^14^,^15^. Additional studies revealed that both early-life and adult adverse socioeconomic circumstances have the potential to alter inflammation status^16^,^17^. Recent studies used a life course approach and identified inflammation markers as (partially) explaining social differences in health^18^,^19^. A vast majority of these studies used C-reactive protein (CRP) as a general proxy to characterise inflammatory status^20^, and some others used alternative inflammatory markers such as interleukin 6 (IL6)^10^,^11^,^12^,^13^, fibrinogen^14^,^15^, or tumour necrosis factor *α* (TNF-*α*)^11^,^13^. Because previous studies relied only on a limited number of inflammatory markers, generalisation of the reported biomarker-specific associations with SEP to the inflammatory burden remains uncertain. In order to capture the natural complexity of inflammatory profiles and their regulation (e.g. pleiotropic effects and partial redundancies across inflammatory markers), we opted here for a more in-depth approach through the use of a wide range of cytokines, chemokines and proliferation factors^21^,^22^,^23^. Multiplex assay platforms enable simultaneous evaluation of a large number of inflammatory circulating markers in small amounts of plasma^24^ and therefore enable a comprehensive evaluation of the role of these markers in our observational context.

Our study aims to investigate how SEP over the life course is likely to be embedded through the inflammatory system. Using proteomic profiles obtained from prospectively collected peripheral blood samples in 268 participants from the Italian component of the European Prospective Investigation into Cancer and Nutrition (EPIC-Italy), we evaluated the association between early, young, and adulthood SEP with 28 markers of inflammation. These were examined individually and jointly as an inflammatory score. We hypothesize that participants with low SEP have a low-grade inflammation response. In addition, we aim to determine whether early life SEP interacts with adult SEP to influence inflammation levels.

## Results

As indicated in Supplementary Table S1, key characteristics of the study population differ from the originating EPIC-Italy population by their age, gender, and recruitment centre: they are older, mostly women recruited in Central Italy (43.3% from Florence). Those differences are all related to the nested case-control design, but no particular differences were observed according their SEP metrics, despite a slightly higher proportion of participants with lower educational level. Detailed description of the study participants is given in Table 1. Irrespective of the SEP indicator, participants in the low SEP group tend to be older, to have a higher BMI, and to exhibit lower smoking prevalence than those in higher SEP categories.

### Inflammatory markers of life course SEP indicators

**Figure 1:**
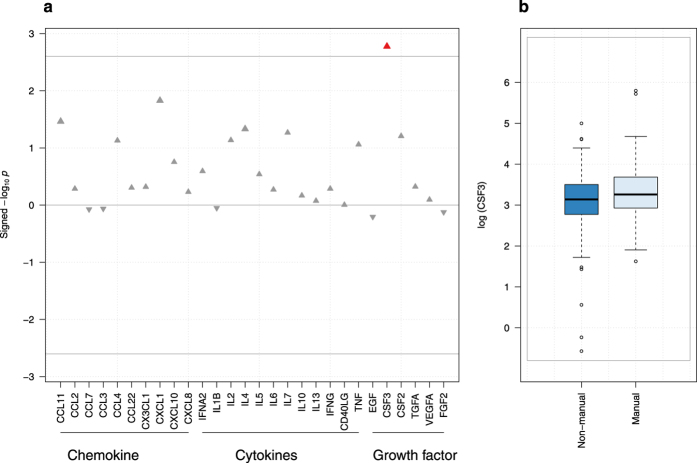
(**a**) Plasma inflammatory factors study of father occupational position. The −log_10_ p-value is signed by the direction of the effect size estimate and is plotted against each of the 28 proteins. The grey line indicates the per-test significance level controlling the FWER at a 5% level. **(b)** Boxplot of log transformed CSF3 (or G-CSF) plasma levels per father occupational position group.

We investigated first associations between each of the 28 inflammatory markers and our three SEP indicators (father’s occupation, education, highest household occupation) separately. For all indicators, the ‘high’ socioeconomic group was used as reference and a positive association therefore indicate an increased level of inflammation ‘low’ socioeconomic group (Table 2A). Inflammatory protein concentrations exhibited general positive associations with father’s occupation, and negative associations with education level and highest household occupation. No association remains significant after correction for multiple testing with both education and highest household occupational position, while one association involving CSF3 levels and father’s occupation survived: lower father’s occupation being associated with higher levels of CSF3 (*β* = 0.29, P = 0.002, Fig. 1). Adjusting for education (model B-1), and additionally for adulthood SEP (model C), the association between CSF3 and early-life SEP, though slightly attenuated, remained significant (P < 0.008). Additional adjustment on the three potential confounders (BMI, smoking, alcohol, see methods) affected only marginally point estimates (Table 3A).

### Inflammatory scores and life course SEP indicators

We defined a discrete inflammatory score (range: [0–27], see methods) measuring overall inflammatory status. The score was found significantly associated exclusively to father’s occupation (Table 2B): the inflammatory score was higher in participants whose father had a low occupational position (*β* = 1.96, P = 0.029); conversely, the inflammatory score was lower in participants with low educational level or low highest household occupation but these associations were not significant (*β* = −1.02, P = 0.26 and *β* = −1.35, P = 0.15 respectively).

The association between inflammatory score and early life SEP (Table 3B) was strengthened by adjusting for education and highest household occupation. As above, further adjustments for potential confounders did not impact our findings. Participant’s education and participant’s highest household occupation became significantly associated with the inflammatory score only when controlling for father’s occupation (model B-1 and model B-2, respectively): the inflammatory score was lower in participants with low educational level or low highest household occupation. Participant’s education and participant’s highest household occupation lost statistical significance when adjusting simultaneously on both SEP indicators in the model (model C).

As a sensitivity analysis, we ran a principal component analysis on the 28 inflammatory markers, and the first principal component (PC1, explaining 35.7% of the variance) was used as an alternative continuous inflammatory score. The use of PC1 as a inflammatory score slightly weakened our results, but these remained consistent: a significant association between PC1 and father’s occupational position, stable after controlling for early life SEP and a non-significant association with participant’s education and highest household occupation, in the opposite direction, that became significant after adjustment on father’s occupation (Table 3C). Because of the observed negative correlation between the inflammatory score and PC1 (*ρ* = −0.89, P < 0.001), a higher PC1 score indicates lower inflammatory score level and effect size estimates have reversed signs.

### Investigating markers of social mobility

**Figure 2:**
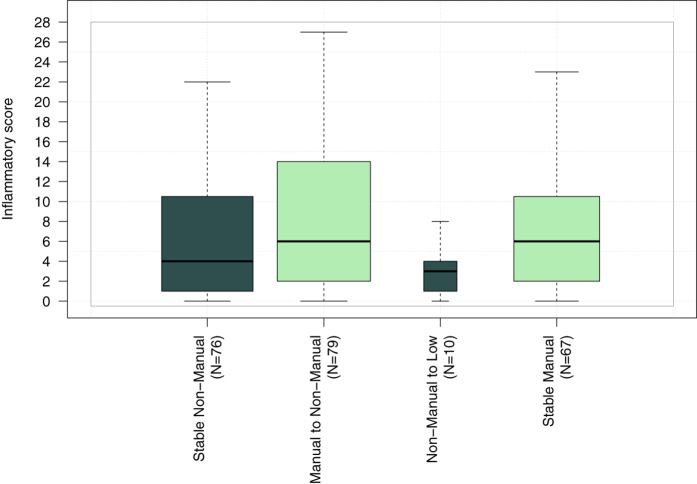
Box-and-whisker plot summarising the distribution of the inflammatory score across the four categories of the social mobility index.

To test the potential for an interaction between early life SEP and the participant’s SEP later in life on the inflammatory score, we modelled the effect of social mobility on the inflammatory score. From the analyses, we identified an increase in the inflammatory score between ‘stable Non-manual’ and ‘Manual to Non-manual’ groups (*β* = 2.38, P = 0.02, Table 4A), suggesting a stimulation of the inflammatory system in participants experiencing upward social mobility (Fig. 2). Results using PC1 as inflammatory score showed a differential inflammatory status between ‘stable Non-manual’ and ‘Manual to Non-manual’ although not significant (*β* = −0.72, P = 0.17, Table 4B).

Sensitivity analyses redefining social mobility as the interaction between father’s occupation and participant’s own education were conducted, and provided similar results, though with stronger associations for the inflammatory score (Supplementary Table S2).

## Discussion

In the present study, we investigated the association between inflammatory markers and SEP at different time points in life using a panel of 28 plasma inflammatory protein concentrations considered separately, or combined into an inflammatory score. We hypothesised that SEP may physiologically be embedded at different time points, and subsequently affect the inflammatory burden. Testing all the 28 markers in relation to the three SEP indicators separately, only one significant association emerged: low father’s occupation was associated with higher CSF3 level. We additionally showed that participants reporting a father with a ‘manual’ occupation had a higher inflammatory score later in life compared to those whose father had a ‘non-manual’ occupation. No significant associations were found between participant’s education and highest household occupation when examined separately. Nevertheless, subsequent analysis of the inflammatory score in a life course context (from childhood to adulthood) indicated that participants with upward social mobility had higher inflammatory scores than those remaining socially advantaged.

To test the robustness of our results to the measure of the inflammatory status we also defined, as an unsupervised alternative, the inflammatory status as the first principal component (PC1) obtained from the 28 inflammatory markers. We identified consistent, though statistically weakened, associations with father’s occupation in our life course models adjusting for participant’s own education and/or highest household occupation. Social mobility analyses using PC1 also provided similar associations, but these did not reach statistical significance. This consistency may at least be partially explained by the strong correlation between our inflammatory score and PC1 (*ρ* = −0.89). Our estimates and conclusions remained markedly stable upon adjustments for behavioral factors (smoking and alcohol consumption, and BMI), hence providing evidence that the inflammatory signal we report as markers of SEP are independent of the potential inflammatory signatures of these factors. We cannot exclude the possibility that other factors may contribute to the mechanisms linking SEP and inflammation. This would include anthropometric and obesity variables such as height, weight, waist and hip circumference and waist to hip ratio. Based on model C, we further adjusted on each of these covariates separately (Supplementary Table S3). We subsequently added physical activity and hormone replacement therapy as potential confounders in the resulting fully adjusted model (Supplementary Table S4). As indicated in these tables, the association between the inflammatory score and father occupational position was robust across all these additional models.

Our study population remains limited in size, which constrained our methodological choices. First, the available variable categories for father’s occupation, participant’s education and highest household occupation were all recoded (binary indicators). The reasoning behind this recoding accounted for 1) conceptual typologies of occupations and educational attainment conferring notions of hierarchy and/or work strain 2) maximising the subgroup samples to achieve sufficient statistical power. Nevertheless, misclassification error may have occurred where incorrect assumptions were made when recoding. As sensitivity analysis we ran all analysis coding professional in the low education class, and results were only marginally changed (Supplementary Table S5). From the socioeconomic indicators available in the EPIC dataset, we selected our 3 measures based on (i) their unambiguous definition, (ii) the clear distinction of the critical life stages each corresponded to, and (iii) their generalisability to other populations. These indicators offer an individual-based measure of socioeconomic experience during the life course, and as such may provide information about individuals’ access to social and economic resources. They may also capture individual factors (e.g. material, behavioral or psychosocial factors) or macro-environmental features (e.g. geographical location) driving the link between the social environment and the inflammatory process. Our results might also be affected by a potential lack of representativeness of our study population which derives from a cancer case control study nested in a cohort. In order to account for potential (case-driven) population bias, we also ran all analyses by restricting the study population to healthy controls. As summarised in Supplementary Table S6, the smaller sample size reduced statistical power, and measures of association were subsequently weakened. Nevertheless, the sign and estimates of the effect sizes were all consistent, and most of the relevant associations were found nominally significant (P < 0.05).

Furthermore, our study population comprises a large proportion of breast cancer prospective cases-control pairs (N = 100 participants). This certainly affects representativeness of the sex ratio within our study population, but may also affect the generalizability of our results, given the higher breast cancer incidence observed in higher SEP classes^25^. The latter might potentially explain the counter-intuitive direction of associations found with young and adulthood SEP indicators separately and inflammation level. Several studies have examined the influence of childhood SEP (as measured by father’s occupation or father’s education) on inflammatory markers levels in adulthood; most of them have shown that lower early life SEP was associated with a greater level of CRP^18^,^26^,^27^, the fibrinogen^18^,^26^,^27^ and the IL-6^18^,^28^. However, little is known about the associations between childhood SEP and other inflammatory markers including other cytokines, chemokines and proliferation factors participating in the inflammatory cascade. Although CSF3 was only found associated while analysing the inflammatory markers individually, our findings demonstrated that father’s occupational position was inversely associated with the inflammatory score, and thus supported the hypothesis of a global impact on the inflammatory system. To our knowledge, a single study investigated relationship between social mobility and inflammatory markers concentration. In this work, Loucks *et al.* reported an elevated level of intercellular adhesion molecule 1 (ICAM-1), tumor necrosis factor receptor 2 (TNFR2) and lipoprotein-associated phospholipase A2(Lp-PLA2) for participants with upward social mobility (low childhood and high adulthood SEP)^18^.

Three models have classically been proposed to explain the link between life course SEP and adult disease^29^. The critical period model postulates that adverse socioeconomic circumstances in early life can permanently modify biological systems with long-term health effects. The accumulation model supposes that accumulation of adversities across the life course has a negative impact on health, while the social mobility framework assumes that SEP may evolve over tim and consider a differential effect of stable or varying SEP trajectories on health and well-being. Our data support both the critical/sensitive period hypothesis, since father’s occupational position had a stronger effect on adulthood inflammatory level and also support the social mobility hypothesis where participants whose SEP increased over time showed higher inflammatory levels. However, our findings do not support the accumulation model.

To our knowledge, the present study is the first to provide a comprehensive analysis of the inflammatory response to socioeconomic experiences in a life course context, using higher resolution profiles. The use of the inflammatory score as a summary measure of the 28 inflammatory markers together with its continuous alternative enabled us to support the hypothesis that early life SEP leads to persistent changes in the inflammation response.

Despite the limitations mentioned above, our study provides evidence supporting the existence of biologically imprinted responses to socioeconomic experiences and trajectories throughout life as formalised in the concept of embodiment^30^. It therefore calls for further work involving larger populations and potentially other ‘–omics’ profiles to explore, at several molecular levels, the mechanisms involved in the biological response to SEP experiences. Although further validation is needed, our results suggest that early SEP, and upward social mobility both have a long term effect on inflammation, that may impact health later in life.

## Methods

### Study population

Our study population arises from the EnviroGenoMarkers (EGM) project, which was initially designed to identify novel biomarkers of non-Hodgkin’s lymphoma and breast cancer risk from multiple ‘–omics’ profiles^23^. We include in this study 268 EGM participants from the Italian component of EPIC^31^, for whom anthropometric, lifestyle, dietary and socioeconomic factors were collected through questionnaires. All participants provided informed consent, and the EPIC study protocol was approved by the review board of the International Agency for Research on Cancer and by all local institutes recruiting participants. The study was conducted in accordance with the approved guidelines. Incident NHL (N = 84) and breast cancer (N = 50) cases were diagnosed between 2 and 13 years after recruitment in EPIC, and were identified through local cancer registries. For each case identified, one random control was selected among all EPIC Italy participants alive and free of cancer at the time of diagnosis of the index case, matched by centre (Turin; Varese; Naples; Ragusa and Florence), gender, date of blood collection (+/−6 months), and age at recruitment (+/−2.5 years). Biosamples underwent inflammatory profiling in two distinct batches including 100 and 34 case/control pairs, respectively. Biosamples were all collected upon inclusion of participants in EPIC Italy. At the time of blood collection, all the participants were free of cancer.

The sample selection strategy is described in Supplementary Figure S1.

### Life course socioeconomic position (SEP)

To preserve power and interpretability, life course SEP factors from the EPIC questionnaire were dichotomised. Childhood SEP was measured by father’s occupation and recoded in the two following categories; i) ‘Manual’ (N = 147) consisting of: unskilled workers (N = 52), skilled workers (N = 59), and farmers (N = 36); and ii) ‘Non-manual’ (N = 87) consisting of: retailers (N = 30), employees (N = 40), and self-employed (N = 17).

Young adulthood SEP was measured through participant’s own education which was dichotomised as i) ‘High’ (above the minimum legal education level, 15 years of age; N = 120): professional (N = 30), upper secondary school (N = 57), and university (N = 33); and ii) ‘Low’ (below the minimum legal education level; N = 147): none (N = 6), primary school (N = 79), lower secondary school (N = 62).

Adulthood SEP was measured using the highest occupational position in the household as defined by either the participant’s own occupation or his/her partner's. It was classified as ‘Manual’ (N = 79) and ‘Non-manual’ (N = 158), following the same categorisation as for father’s occupation. Characteristics of the 230 participants with full SEP information are summarised in Table 1.

### Laboratory analyses

For each participant, a blood sample was collected at enrollment and, within two hours, subsequently processed for the isolation of buffy coats and other fractions, which were placed in cold storage (liquid nitrogen). A panel of inflammation-related proteins (N = 32) was measured using the MILLIPLEX HCYTOMAG-60K and HSCYTMAG-60SK kits (Millipore, Billerca, MA), according to the manufacturer’s protocol. As detailed in Supplementary table S7, measurements included 10 chemokines, 12 cytokines, and 6 growth factors. Four analytes (IL-12, IL1-RA, sIL2-RA and Flt3ligand) were excluded from further statistical analyses due to a high rate of non-detects (>75%). The lower limits of detection (LOD) are also reported in Supplementary Table S7.

### Inflammatory measures

Initially, we considered all protein levels separately. As depicted in Supplementary Figure S2, protein concentrations exhibit a strong pairwise correlation. PCA of these 28 inflammatory markers concentration showed that 20 PCs explained more than 95% of the total variation seen in the data set (Supplementary Figure S3). The multiple testing corrected significance level accounts for the correlation in the data and is defined as P = 0.05/20 (P = 0.0025).

Finally, we defined an inflammatory score from the 28 protein concentrations. We assumed, as already reported^32^ and in accordance with the theory of a global wear-and-tear of the organism due to stressful events^33^, a global positive association between inflammation and decreasing SEP. For each protein, we defined a dichotomised indicator: ‘high concentration’ = 1, and ‘low concentration’ = 0 based on the highest quartile of the log-transformed concentrations, and summed these across the 28 proteins^34^. As a continuous and hypothesis-free alternative, we used the first principal component (explaining 35.7% of the total variance).

### Statistical Model

Statistical analyses were performed using R v3.1.2^35^. Protein levels below the LOD were imputed based on a maximum likelihood estimation method using the correlation structure within the data to draw the missing values^36^. In all analyses, levels of proteins were log-transformed to normalise their distributions.

As proposed elsewhere^37^, the per-protein analyses were based on a linear mixed model assuming technically-induced variance across microtiter plates induced a systematic shift in the concentration measures. We included a random intercept in the model, noted 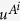, and representing the shift associated to *A*^*i*^, the identifier of the plate on which sample *i* was assayed. For sample *i* the model is defined as follows:


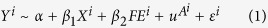


where *Y*^*i*^ represents the inflammatory measure in participant i, *α* is the intercept, *ε*^*i*^ is the residual error, *X*^*i*^ is the binary SEP indicator observed in that same participant (where the highest class is used as the reference category) whose effect is measured by the regression coefficient *β*_1_, and *FE*^*i*^ is a matrix of fixed effect observations and corresponding regression coefficients are compiled in the vector *β*_2_. Fixed effect covariates include the case-control matching criteria (age, gender, and centre, recoded in three categories: North, Central, and South Italy) and batch. To account for the case-control design of EGM, we also included two binaries indicators of whether a participant is a prospective breast cancer or lymphoma case.

The inflammatory score analysis and PCA were based on ‘de-noised’ protein concentrations as obtained from the above linear mixed model by subtracting the random effect estimates from the observed levels. As such, these measures are implicitly corrected for technically-induced variation, and were analysed using a linear model corresponding to (1) setting the random intercept term to zero.

### Life course analyses

For the different measures of inflammatory status described above, we used the same benchmark model, and, to mimic life course experiences, we sequentially adjusted for the following chronologically ordered proxies for early life, young adulthood, and adulthood SEP indicators; resulting in four time-sequenced models:

(A) Age, gender, case-control status, batch, centre and father’s occupation;

(B-1) Model A + education;

(B-2) Model A + highest household occupation;

(B) Model B-1 + highest household occupation;

A fully adjusted model was subsequently built upon model C including body mass index (BMI, kg/m2), smoking status (categorical: current, former, and never smoker), and alcohol consumption (g/day) as three potential SEP-driven factors.

To model social mobility, we generalized the linear model used for our inflammatory score by introducing a multiplicative interaction term for father’s occupation and highest household occupation, hence defining 4 classes:‘Stable Non-manual’ (reference); ‘Manual to Non- manual’; ‘Non-manual to Manual’; and ‘Stable Manual’.

Several sensitivity analyses were conducted. First, to preserve power, we dichotomized SEP indicators, while, especially for education, three categories would have been more relevant. In particular, based on the minimal legal education level in Italy our choice to include ‘professionals’ in the ‘high’ education category may be arguable. We ran all the analyses coding professional in the low education class.

The data we used arose from a case-control study nested in a prospective cohort. To assess the potential for reverse causation, we ran all analyses by restricting the study population to healthy controls.

Social mobility was modelled by introducing a multiplicative interaction term for father’s occupation and participant’s education.

## Additional Information

**How to cite this article**: Castagné, R. *et al.* A life course approach to explore the biological embedding of socioeconomic position and social mobility through circulating inflammatory markers. *Sci. Rep.* **6**, 25170; doi: 10.1038/srep25170 (2016).

## Supplementary Material

Supplementary Information

## Figures and Tables

**Table 1:** Summary characteristics of the study population.

	Father’s occupational position (N = 234)			Participant’s education (N = 267)			Household’s highest occupational position (N = 237)			Participants with complete data (N = 230)
	Non Manual	Manual	P-value	High	Low	P-value	Non Manual	>Manual	P-value	N% or mean (sd)
N (%)	87 (37.2)	147 (62.8)		120 (44.9%)	147 (55.1)		158 (66.7)	79 (33.3)		230 (100)
Age, yo, mean (SD)	52.6 (7.7)	54.2 (8.3)	0.14	50.9 (7.4)	55.3 (8.0)	<0.0001	53.0 (7.9)	55.0 (8.2)	0.07	53.7 (8.0)
Gender, N (%)										
Women	66 (75.9)	103 (70.1)	0.42	87 (72.5)	113 (76.9)	0.50	112 (70.9)	57 (72.2)	0.96	166 (72.2)
Men	21 (24.1)	44 (29.9)		33 (27.5)	34 (23.1)		46 (29.1)	22 (27.8)		64 (27.8)
Breast case, N (%)	20 (23.0)	23 (15.6)	0.22	26 (21.7)	24 (16.3)	0.34	34 (21.5)	8 (10.1)	0.05	41 (17.8)
NHL case, N (%)	29 (33.3)	45 (30.6)	0.77	34 (28.3)	50 (34.0)	0.39	55 (34.8)	22 (27.9)	0.35	74 (32.2)
Center*, N (%)										
South	4 (4.5)	18 (12.2)	0.02	23 (19.2)	24 (16.3)	0.02	12 (7.6)	9 (11.4)	0.26	20 (8.7)
Central	50 (57.5)	60 (40.8)		61 (50.8)	55 (37.4)		81 (51.3)	32 (40.5)		109 (47.3)
North	33 (38.0)	69 (47.0)		36 (30.0)	68 (46.2)		65 (41.1)	38 (48.1)		101 (44.0)
Phase, N (%)										
Phase 1	66 (75.9)	109 (74.1)	0.89	97 (80.8)	103 (70.1)	0.06	125 (79.1)	52 (65.8)	0.04	172 (74.8)
Phase 2	21 (24.1)	38 (25.9)		23 (19.2)	44 (29.9)		33 (20.9)	27 (34.2)		58 (25.2)
Body mass index, mean (SD)	25.3 (3.4)	26.0 (4.0)	0.10	24.8 (3.1)	26.6 (3.7)	0.00	25.3 (3.3)	26.6 (3.8)	0.01	25.8 (3.5)
Smoking status, N (%)										
Never	38 (43.7)	79 (53.7)	0.24	47 (39.2)	82 (55.8)	0.03	71 (44.9)	45 (57.0)	0.18	115 (50.0)
Former	28 (32.2)	34 (23.1)		39 (32.5)	35 (23.8)		46 (29.1)	17 (21.5)		62 (27.0)
Current	21 (24.1)	33 (22.4)		34 (28.3)	30 (20.4)		41 (25.9)	16 (20.2)		53 (23.0)
Missing	0 (0.0)	1 (0.7)		0 (0.0)	0 (0.0)		0 (0.0)	1 (1.3)		0 (0.0)
Grams alcohol/day, mean (SD)	14.4 (19.7)	10.8 (14.7)	0.13	12.0 (15.4)	11.0 (16.1)	0.60	12.1 (15.2)	13.0 (19.7)	0.72	12.2 (16.8)
Social mobility										
stable Non-manual										76 (33.0)
Manual to Non-manual										79 (34.3)
Non-Manual to Manual										10 (4.3)
stable Manual										65 (28.3)

Population features are also summarized for each SEP category.

Counts and percentages are reported for categorical variable, and means and standard deviations for continous variables.

P-value for difference was calculated using the chi-squared test for categorical variables and the student’s t-test for continuous variables.

^*^North: Turin & Varese; Central: Florence; South: Naples & Ragusa.

**Table 2:** Results for the 28 plasma levels of inflammatory factors and each of the three SEP factors.

	Father’s occupational position			Participant’s education			Household’s highest occupation		
	N	*β* (SE)	P-value	N	*β* (SE)	P-value	N	*β* (SE)	P-value
(A) Inflammatory markers									
CCL11	234	0.18 (0.09)	0.034	267	−0.05 (0.08)	0.546	237	−0.02 (0.09)	0.805
CCL2	234	0.03 (0.05)	0.518	267	−0.05 (0.05)	0.310	237	−0.03 (0.05)	0.544
CCL7	234	−0.05 (0.25)	0.846	267	−0.36 (0.23)	0.117	237	−0.47 (0.26)	0.072
CCL3	234	−0.03 (0.18)	0.868	267	−0.27 (0.17)	0.119	237	−0.31 (0.19)	0.103
CCL4	234	0.23 (0.13)	0.074	267	−0.12 (0.12)	0.292	237	−0.14 (0.13)	0.280
CCL22	234	0.07 (0.11)	0.497	267	−0.1 (0.10)	0.310	237	−0.03 (0.11)	0.799
CX3CL1	234	0.13 (0.19)	0.480	267	−0.38 (0.17)	0.026	237	−0.2 (0.19)	0.296
CXCL1	234	0.20 (0.08)	0.015	267	−0.05 (0.08)	0.506	237	0.02 (0.09)	0.823
CXCL10	234	0.10 (0.07)	0.176	267	−0.08 (0.07)	0.244	237	0.00 (0.07)	0.962
CXCL8	234	0.07 (0.13)	0.589	267	−0.06 (0.13)	0.613	237	−0.09 (0.14)	0.515
IFNA2	234	0.51 (0.45)	0.255	267	−0.07 (0.42)	0.876	237	−0.27 (0.46)	0.560
IL1B	234	−0.04 (0.27)	0.891	266	−0.06 (0.25)	0.799	237	0.08 (0.28)	0.763
IL2	234	0.31 (0.17)	0.073	267	−0.12 (0.17)	0.481	237	0.32 (0.18)	0.078
IL4	234	0.40 (0.20)	0.047	266	0.05 (0.18)	0.793	237	0.12 (0.21)	0.547
IL5	234	0.17 (0.16)	0.289	267	0.05 (0.15)	0.755	237	0.21 (0.17)	0.222
IL6	234	0.13 (0.22)	0.537	267	0.02 (0.21)	0.934	237	0.24 (0.22)	0.279
IL7	234	0.19 (0.10)	0.054	267	−0.15 (0.09)	0.113	237	0.03 (0.10)	0.726
IL10	234	0.10 (0.25)	0.681	267	−0.02 (0.24)	0.929	237	0.05 (0.26)	0.858
IL13	234	0.05 (0.24)	0.843	267	−0.19 (0.23)	0.413	237	0.15 (0.25)	0.554
IFNG	234	0.18 (0.28)	0.517	267	−0.23 (0.28)	0.403	237	−0.13 (0.30)	0.665
CD40LG	234	0.00 (0.09)	0.993	267	−0.04 (0.09)	0.693	237	0.00 (0.09)	0.970
TNF	234	0.13 (0.08)	0.087	267	0.03 (0.08)	0.686	237	0.08 (0.08)	0.345
EGF	234	−0.13 (0.27)	0.625	267	−0.58 (0.26)	0.024	237	−0.64 (0.28)	0.024
CSF3*	234	0.29 (0.09)	0.002	267	0.12 (0.09)	0.154	237	0.15 (0.10)	0.122
CSF2	234	0.26 (0.14)	0.062	267	−0.16 (0.13)	0.236	237	0.14 (0.15)	0.338
TGFA	234	0.21 (0.29)	0.476	267	−0.19 (0.27)	0.491	237	−0.40 (0.30)	0.184
VEGFA	234	0.06 (0.25)	0.804	267	−0.5 (0.23)	0.031	237	−0.48 (0.26)	0.062
FGF2	234	−0.07 (0.24)	0.755	267	−0.34 (0.22)	0.127	237	−0.63 (0.24)	0.010
(B) Inflammatory scores									
Inflammatory score	230	1.96 (0.89)	0.029	230	−1.02 (0.91)	0.261	230	−1.35 (0.93)	0.151
PC1	230	−0.60 (0.45)	0.182	230	0.66 (0.45)	0.140	230	0.51 (0.46)	0.268

Results are also presented for the inflammatory score and the first PC.

Model adjusted on age, gender, lymphoma case-control status, breast cancer case-control status, phase and center.

^*^Significant association with father’s occupational position after multiple testing correction (P < 0.0025).

**Table 3:** Life course multiple regression analyses for father’s occupational position and inflammatory status.

Variables	Levels	Model A		Model B-1		Model B-2		Model C		Fully Adjusted Model	
		*β* (SE)	P-value	*β* (SE)	P-value	*β* (SE)	P-value	*β* (SE)	P-value	*β* (SE)	P-value
(A) Plasma concentration of CSF3											
Father’s occupational position	Manual	0.29 (0.09)	0.002	0.27 (0.10)	0.008	0.29 (0.10)	0.004	0.28 (0.10)	0.008	0.28 (0.10)	0.007
Participant’s education	Low			0.03 (0.10)	0.733	–	–	0.04 (0.11)	0.742	0.02 (0.12)	0.868
Household’s highest occupation	Manual					0.01 (0.10)	0.916	−0.01 (0.11)	0.957	−0.02 (0.11)	0.862
BMI										0.02 (0.01)	0.230
Smoking status	Former									−0.01 (0.12)	0.917
	Current									−0.01 (0.12)	0.608
Alcohol										0.001 (0.003)	0.644
(B) Inflammatory score											
Father’s occupational position	Manual	1.96 (0.89)	0.029	2.88 (0.97)	0.003	2.64 (0.93)	0.005	3.08 (0.98)	0.002	2.93 (1.00)	0.004
Participant’s education	Low			−2.22 (0.98)	0.024	–	–	−1.54 (1.08)	0.156	−1.5 (1.10)	0.174
Household’s highest occupation	Manual					−2.22 (0.97)	0.023	−1.56 (1.07)	0.149	−1.49 (1.09)	0.174
BMI										−0.07 (0.13)	0.617
Smoking status	Former									−0.62 (1.16)	0.594
	Current									−0.57 (1.16)	0.621
Alcohol										−0.02 (0.03)	0.433
(C) Principal component 1											
Father’s occupational position	Manual	−0.60 (0.45)	0.182	−1.05 (0.49)	0.031	−0.84 (0.47)	0.074	−1.10 (0.49)	0.026	−1.06 (0.50)	0.034
Participant’s education	Low			1.10 (0.49)	0.025	–	–	0.93 (0.54)	0.088	0.95 (0.55)	0.086
Household’s highest occupation	Manual					0.79 (0.49)	0.104	0.39 (0.54)	0.466	0.40 (0.55)	0.462
BMI										−0.01 (0.07)	0.856
Smoking status	Former									0.27 (0.58)	0.639
	Current									0.48 (0.58)	0.411
Alcohol										0.01 (0.01)	0.637

Results are presented for plasma concentration of CSF3 **(A)**, and for the inflammatory score **(B)** and the first PC **(C)**. Estimates are based on 230 participants with full SEP and lifestyle information.

**Table 4:** Multiple regression analyses of social mobility through the interaction term between father’s occupation and participant highest household position.

Variables	Social mobility		
	*β*	SE	P-value
(A) Inflammatory score			
Intercept (stable Non-manual)	8.40	3.18	0.009
Manual to Non-manual	2.38	1.04	0.023
Non-Manual to Manual	−3.36	2.16	0.122
stable Manual	0.42	1.11	0.705
(B) Principal component 1			
Intercept (stable Non-manual)	−1.89	1.59	0.236
Manual to Non-manual	−0.72	0.52	0.170
Non-Manual to Manual	1.33	1.08	0.222
stable Manual	−0.05	0.56	0.933

Results are presented for the inflammatory score **(A)** and the first PC **(B)**.

## References

[b1] Mackenbach J. P. *et al.* Socioeconomic Inequalities in Health in 22 European Countries. N. Engl. J. Med. 358, 2468–2481 (2008). 18525043 10.1056/NEJMsa0707519

[b2] Mackenbach J. P. Health inequalities: Europe in profile (Produced by COI for the Department of Health, 2006).

[b3] Marmot M. *et al.* Closing the gap in a generation: health equity through action on the social determinants of health. The Lancet 372, 1661–1669 (2008). 10.1016/S0140-6736(08)61690-6 18994664

[b4] Stringhini S. *et al.* Health Behaviours, Socioeconomic Status, and Mortality: Further Analyses of the British Whitehall II and the French GAZEL Prospective Cohorts. PLos Med. 8, e1000419 (2011). 21364974 10.1371/journal.pmed.1000419 PMC3043001

[b5] Gallo V. *et al.* Social Inequalities and Mortality in Europe – Results from a Large Multi-National Cohort. PLos One 7, e39013 (2012). 22848347 10.1371/journal.pone.0039013 PMC3405077

[b6] Blane D., Kelly-Irving M., d’Errico A., Bartley M. & Montgomery S. Social-biological transitions: how does the social become biological? Longitud. Life Course Stud. 4, 136–146 (2013).

[b7] McEwen B. S. Physiology and Neurobiology of Stress and Adaptation: Central Role of the Brain. Physiol. Rev. 87, 873–904 (2007). 17615391 10.1152/physrev.00041.2006

[b8] Yang E. V. & Glaser R. Stress-induced immunomodulation and the implications for health. Int. Immunopharmacol. 2, 315–324 (2002). 11811934 10.1016/s1567-5769(01)00182-5

[b9] Scrivo R., Vasile M., Bartosiewicz I. & Valesini G. Inflammation as “common soil” of the multifactorial diseases. Autoimmun. Rev. 10, 369–374 (2011). 21195808 10.1016/j.autrev.2010.12.006

[b10] Ranjit N. *et al.* Socioeconomic position, race/ethnicity, and inflammation in the multi-ethnic study of atherosclerosis. Circulation 116, 2383–2390 (2007). 18025402 10.1161/CIRCULATIONAHA.107.706226

[b11] Koster A. *et al.* Association of inflammatory markers with socioeconomic status. J. Gerontol. A. Biol. Sci. Med. Sci. 61, 284–290 (2006). 16567379 10.1093/gerona/61.3.284

[b12] Gruenewald T. L., Cohen S., Matthews K. A., Tracy R. & Seeman T. E. Association of socioeconomic status with inflammation markers in black and white men and women in the Coronary Artery Risk Development in Young Adults (CARDIA) study. Soc. Sci. Med. 69, 451–459 (2009). 19524346 10.1016/j.socscimed.2009.05.018 PMC2747365

[b13] Fraga S. *et al.* Association of socioeconomic status with inflammatory markers: a two cohort comparison. Prev. Med. 71, 12–19 (2015). 25482420 10.1016/j.ypmed.2014.11.031

[b14] Panagiotakos D. B. *et al.* The association between educational status and risk factors related to cardiovascular disease in healthy individuals: The ATTICA study. Ann. Epidemiol. 14, 188–194 (2004). 15036222 10.1016/S1047-2797(03)00117-0

[b15] Steinvil A. *et al.* Relation of educational level to inflammation-sensitive biomarker level. Am. J. Cardiol. 102, 1034–1039 (2008). 18929705 10.1016/j.amjcard.2008.05.055

[b16] Kelly-Irving M., Mabile L., Grosclaude P., Lang T. & Delpierre C. The embodiment of adverse childhood experiences and cancer development: potential biological mechanisms and pathways across the life course. Int. J. Public Health 58, 3–11 (2013). 22588310 10.1007/s00038-012-0370-0

[b17] Ben-Shlomo Y. & Kuh D. A life course approach to chronic disease epidemiology: conceptual models, empirical challenges and interdisciplinary perspectives. Int. J. Epidemiol. 31, 285–293 (2002). 11980781

[b18] Loucks E. B. *et al.* Life course socioeconomic position is associated with inflammatory markers: the Framingham Offspring Study. Soc. Sci. Med. 71, 187–195 (2010). 20430502 10.1016/j.socscimed.2010.03.012 PMC2895737

[b19] Stringhini S. *et al.* Association of lifecourse socioeconomic status with chronic inflammation and type 2 diabetes risk: the Whitehall II prospective cohort study. PLos Med. 10, e1001479 (2013). 23843750 10.1371/journal.pmed.1001479 PMC3699448

[b20] Ansar W. & Ghosh S. C-reactive protein and the biology of disease. Immunol. Res. 56, 131–142 (2013). 23371836 10.1007/s12026-013-8384-0

[b21] Leng S. X. *et al.* ELISA and multiplex technologies for cytokine measurement in inflammation and aging research. J. Gerontol. A. Biol. Sci. Med. Sci. 63, 879–884 (2008). 18772478 10.1093/gerona/63.8.879 PMC2562869

[b22] Moncunill G., Aponte J. J., Nhabomba A. J. & Dobaño C. Performance of multiplex commercial kits to quantify cytokine and chemokine responses in culture supernatants from Plasmodium falciparum stimulations. PLos One 8, e52587 (2013). 23300981 10.1371/journal.pone.0052587 PMC3534665

[b23] Hebels D. G. A. J. *et al.* Performance in omics analyses of blood samples in long-term storage: opportunities for the exploitation of existing biobanks in environmental health research. Environ. Health Perspect. 121, 480–487 (2013). 23384616 10.1289/ehp.1205657 PMC3620742

[b24] Chaturvedi A. K. *et al.* Evaluation of multiplexed cytokine and inflammation marker measurements: a methodologic study. Cancer Epidemiol. Biomark. Prev. Publ. Am. Assoc. Cancer Res. Cosponsored Am. Soc. Prev. Oncol. 20, 1902–1911 (2011). 10.1158/1055-9965.EPI-11-0221 PMC3400264 21715603

[b25] Klassen A. C. & Smith K. C. The enduring and evolving relationship between social class and breast cancer burden: a review of the literature. Cancer Epidemiol. 35, 217–234 (2011). 21470929 10.1016/j.canep.2011.02.009

[b26] Lawlor D. A., Smith G. D., Rumley A., Lowe G. D. O. & Ebrahim S. Associations of fibrinogen and C-reactive protein with prevalent and incident coronary heart disease are attenuated by adjustment for confounding factors. British Women’s Heart and Health Study. Thromb. Haemost. 93, 955–963 (2005). 15886815 10.1160/TH04-12-0805

[b27] Pollitt R. A. *et al.* Early-life and adult socioeconomic status and inflammatory risk markers in adulthood. Eur. J. Epidemiol. 22, 55–66 (2007). 17225957 10.1007/s10654-006-9082-1

[b28] Mendall M. A. *et al.* Relation of serum cytokine concentrations to cardiovascular risk factors and coronary heart disease. Heart 78, 273–277 (1997). 9391290 10.1136/hrt.78.3.273 PMC484930

[b29] Ben-Shlomo Y. & Kuh D. A life course approach to chronic disease epidemiology: conceptual models, empirical challenges and interdisciplinary perspectives. Int. J. Epidemiol. 31, 285–293 (2002). 11980781

[b30] Krieger N. Embodiment: a conceptual glossary for epidemiology. J. Epidemiol. Community Health 59, 350–355 (2005). 15831681 10.1136/jech.2004.024562 PMC1733093

[b31] Palli D. *et al.* A molecular epidemiology project on diet and cancer: the EPIC-Italy Prospective Study. Design and baseline characteristics of participants. Tumori 89, 586–593 (2003). 14870823 10.1177/030089160308900602

[b32] Fraga S. *et al.* Association of socioeconomic status with inflammatory markers: a two cohort comparison. Prev. Med. 71, 12–19 (2015). 25482420 10.1016/j.ypmed.2014.11.031

[b33] Seeman T. E., Singer B. H., Rowe J. W., Horwitz R. I. & McEwen B. S. Price of adaptation-allostatic load and its health consequences. MacArthur studies of successful aging. Arch. Intern. Med. 157, 2259–2268 (1997). 9343003

[b34] Juster R.-P., McEwen B. S. & Lupien S. J. Allostatic load biomarkers of chronic stress and impact on health and cognition. Neurosci. Biobehav. Rev. 35, 2–16 (2010). 19822172 10.1016/j.neubiorev.2009.10.002

[b35] Development Core Team, R: A language and environment for statistical computing. R Foundation for Statistical Computing, Vienna, Austria. ISBN 3-900051-07-0 (2005).

[b36] Lubin J. H. *et al.* Epidemiologic evaluation of measurement data in the presence of detection limits. Environ. Health Perspect. 112, 1691–1696 (2004). 15579415 10.1289/ehp.7199 PMC1253661

[b37] Chadeau-Hyam M. *et al.* Prediagnostic transcriptomic markers of Chronic lymphocytic leukemia reveal perturbations 10 years before diagnosis. Ann. Oncol. 25, 1065–1072 (2014). 24558024 10.1093/annonc/mdu056 PMC4366593

