# Supplementary material for: A life course approach to explore the biological embedding of socioeconomic position and social mobility through circulating inflammatory markers
Source: Sci Rep. 2016 Apr 27;6:25170. doi: 10.1038/srep25170 (PMC4846829; doi:10.1038/srep25170)
Supplement: Supplementary Information [file srep25170-s1.pdf]

A life course approach to explore the biological embedding of socioeconomic position and social mobility through circulating inflammatory markers

Raphaële Castagné[1,2], Cyrille Delpierre[2], Michelle Kelly-Irving[2], Gianluca Campanella[1], Florence Guida[1], Vittorio Krogh[3], Domenico Palli[4], Salvatore Panico[5], Carlotta Sacerdote[6], Rosario Tumino[7], Soterios Kyrtopoulos[8], Fatemeh Saberi Hosnijeh[9], Thierry Lang[2], Roel Vermeulen[1, 9], Paolo Vineis[1, 10,11], Silvia Stringhini[12], and Marc Chadeau-Hyam[1, 9, 11, *]

[1]Department of Epidemiology and Biostatistics, School of Public Health, Imperial College London, London, UK
[2]INSERM, UMR1027, UniversitéToulouse III-Paul Sabatier, 31000 Toulouse, France
[3]Fondazione IRCCS- Instituto Nazionale dei Tumori, Milan, Italy
[4]Istituto per lo Studio e la Prevenzione Oncologica (ISPO Toscana), Florence, Italy
[5]Department of Clinical Medicine and Surgery, University of Naples Federico II, Naples, Italy
[6]Piedmont Reference Centre for Epidemiology and Cancer Prevention (CPO Piemonte), Turin, Italy
[7]Cancer registry and Histopathology Unit, Azienda Ospedaliera 'Civile-M.P.Arezzo', Ragusa, Italy
[8]National Hellenic Research Foundation, Institute of Biology, Pharmaceutical Chemistry and Biotechnology, Athens, Greece
[9]Institute for Risk Assessment, Division of Environmental Epidemiology, Utrecht University, Utrecht, the Netherlands
[10]HuGeF, Human Genetics Foundation, Torino, Italy
[11]MRC-PHE Centre for Environment and Health, Imperial College, London, London, UK
[12]Institute of Social and Preventive Medicine, Lausanne University Hospital, Lausanne, Switzerland
[*]m.chadeau@imperial.ac.uk

Supplemental Material

Supplementary Table 1. Baseline characteristics and life course socioeconomic position for the full EPIC-Italy population (N=47 749), and for the study population (N=268).

		All EPIC-Italy		Study Population		
		N	% or mean(sd)	N	% or mean(sd)	P-value*
Gender	Men	15 171	31.8%	68	25.4%	2.85E-02
	Women	32 578	68.2%	200	74.6%	
Age (yo)	All	47 749	50.5 (7.9)	268	53.3 (8.0)	2.99E-08
Center	Florence	13 597	28.5%	116	43.3%	3.39E-13
	Varese	12 083	25.3%	88	32.8%	
	Ragusa	6 403	13.4%	22	8.2%	
	Turin	10 604	22.2%	16	6.0%	
	Naples	5 062	10.6%	26	9.7%	
Father's occupational position	Non Manual	14 133	29.6%	87	32.5%	3.28E-01
	Manual	27 503	57.6%	147	54.9%	
	Missing	6 113	12.8%	34	12.6%	
Participant's education	High	23 734	49.7%	120	44.8%	6.64E-02
	Low	23 050	48.3%	147	54.8%	
	Missing	965	2.0%	1	0.4%	
Household's highest occupational position	Non Manual	28 167	59.0%	158	59.0%	9.40E-01
	Manual	13 799	28.9%	79	29.5%	
	Missing	5 783	12.1%	31	11.5%	
Body mass index (kg/m2)	All	47 219	26.0 (4.1)	266	25.8 (3.6)	3.16E-01
	Missing	530	-	2	-	
Smoking	Never	21 589	45.2%	129	48.1%	5.69E-01
	Former	12 710	26.6%	74	27.6%	
	Current	12 557	26.3%	64	23.9%	
	Missing	893	1.9%	1	0.4%	
Grams alcohol/day	All	46839	12.6 (16.8)	267	11.4 (16.1)	2.34E-01
	Missing	9 010	-	1	-	

* P-values were calculated using a chi-squared test for categorical variables, and a Student's T-test for continuous variables

Supplementary Table 2. Multiple regression analyses of social mobility through the interaction term between father's occupation and participant highest household position. Results are presented for the inflammatory score **(A)** and the first PC **(B)**.

	Social mobility with participant's education		
**(A) Inflammatory score**			
**Variables**	β	**SE**	**P-value**
Intercept (Non-Manual – High)	7.22	3.27	0.028
Manual to High	3.51	1.31	0.008
Non Manual to Low	-1.36	1.54	0.378
Manual – Low	0.76	1.07	0.479
**(B) Principal component 1**			
**Variables**	β	**SE**	**P-value**
Intercept (Non-Manual – High)	-1.36	1.63	0.405
Manual to High	-1.07	0.65	0.104
Non Manual to Low	1.08	0.77	0.161
Manual – Low	0.05	0.53	0.928

Supplementary Table 3. Model C with additional adjustment for anthropometric and obesity metrics. Results are presented for the inflammatory score **(A)** and the first PC **(B)**.

		Model C + BMI (N=221)		Model C + Waist Circumference (N=221)		Model C + Hip Circumference (N=221)		Model C + Waist to Hip Ratio (N=221)		Model C + Weight (N=221)		Model C + Height (N=221)	
**(A) Inflammatory score**													
**Variables**	**Levels**	**β (SE)**	**P-value**	**β (SE)**	**P-value**	**β (SE)**	**P-value**	**β (SE)**	**P-value**	**β (SE)**	**P-value**	**β (SE)**	**P-value**
Father's occupational position	Manual	3.06 (1)	0.002	3.05 (0.99)	0.002	3.06 (0.99)	0.002	3.07 (0.99)	0.002	3.03 (0.99)	0.003	3.05 (0.99)	0.002
Participant's education	Low	-1.58 (1.12)	0.160	-1.5 (1.12)	0.179	-1.57 (1.11)	0.160	-1.56 (1.11)	0.163	-1.54 (1.11)	0.168	-1.72 (1.11)	0.123
Household's highest occupation	Manual	-1.92 (1.12)	0.087	-1.89 (1.11)	0.092	-1.9 (1.12)	0.091	-1.95 (1.11)	0.082	-1.92 (1.11)	0.085	-2.07 (1.11)	0.065
BMI		-0.04 (0.13)	0.771	-	-	-	-	-	-	-	-	-	-
Waist Circumference		-	-	-0.04 (0.05)	0.37	-	-	-	-	-	-	-	-
Hip Circumference		-	-	-	-	-0.04 (0.06)	0.509	-	-	-	-	-	-
Waist Hip Ratio		-	-	-	-	-	-	-3.93 (6.36)	0.538	-	-	-	-
Weight		-	-	-	-	-	-	-	-	-0.04 (0.05)	0.38	-	-
Height		-	-	-	-	-	-	-	-	-	-	-7.92 (7.14)	0.268
**(B) Principal component 1**													
**Variables**	**Levels**	**β (SE)**	**P-value**	**β (SE)**	**P-value**	**β (SE)**	**P-value**	**β (SE)**	**P-value**	**β (SE)**	**P-value**	**β (SE)**	**P-value**
Father's occupational position	Manual	-1.07 (0.5)	0.034	-1.06 (0.5)	0.036	-1.07 (0.5)	0.035	-1.07 (0.5)	0.035	-1.06 (0.5)	0.037	-1.05 (0.5)	0.037
Participant's education	Low	0.99 (0.57)	0.081	0.94 (0.56)	0.098	0.97 (0.56)	0.087	0.95 (0.56)	0.095	0.95 (0.56)	0.092	1.03 (0.56)	0.069
Household's highest occupation	Manual	0.5 (0.57)	0.377	0.47 (0.56)	0.409	0.48 (0.56)	0.397	0.48 (0.56)	0.394	0.48 (0.56)	0.397	0.55 (0.56)	0.333
BMI		-0.02 (0.07)	0.776	-	-	-	-	-	-	-	-	-	-
Waist Circumference		-	-	0.01 (0.03)	0.625	-	-	-	-	-	-	-	-
Hip Circumference		-	-	-	-	0 (0.03)	0.899	-	-	-	-	-	-
Waist Hip Ratio		-	-	-	-	-	-	1.69 (3.22)	0.599	-	-	-	-
Weight		-	-	-	-	-	-	-	-	0.01 (0.02)	0.696	-	-
Height		-	-	-	-	-	-	-	-	-	-	4.38 (3.6)	0.226

Supplementary Table 4. Fully adjusted model additionally controlling either for the Cambridge index for physical activity or hormone replacement therapy

		Fully Adjusted Model + Physical Activity (N=228)		Fully Adjusted Model + Hormone therapy replacement (N=229)	
**(A) Inflammatory score**					
**Variables**	**Levels**	**β(SE)**	**P-value**	**β(SE)**	**P-value**
Father's occupational position	Manual	2.87 (1.00)	0.004	3.13 (1.00)	0.002
Participant's education	Low	-1.57 (1.10)	0.154	-1.87 (1.11)	0.094
Household's highest occupation	Manual	-1.58 (1.09)	0.151	-1.49 (1.1)	0.177
BMI		-0.05 (0.13)	0.686	-0.03 (0.13)	0.817
Smoking status	Former	-0.77 (1.17)	0.515	0.06 (1.1)	0.957
	Current	-0.33 (1.17)	0.776	-0.3 (1.18)	0.801
Alcohol		-0.03 (0.03)	0.342	-0.01 (0.03)	0.832
Physical activity (Cambridge Index)	Moderately inactive	-0.43 (1.15)	0.710	-	-
	Moderately active	-2.35 (1.35)	0.084	-	-
	Active	-1.27 (1.61)	0.428	-	-
Hormone replacement therapy*	Yes	-	-	-1.54 (1.18)	0.192
**(B) Principal component 1**					
**Variables**	**Levels**	**β(SE)**	**P-value**	**β(SE)**	**P-value**
Father's occupational position	Manual	-1.03 (0.5)	0.041	-1.12 (0.50)	0.026
Participant's education	Low	0.97 (0.55)	0.081	1.04 (0.56)	0.062
Household's highest occupation	Manual	0.38 (0.55)	0.495	0.42 (0.55)	0.441
BMI		-0.02 (0.07)	0.786	-0.02 (0.07)	0.718
Smoking status	Former	0.37 (0.59)	0.530	0.05 (0.55)	0.930
	Current	0.39 (0.58)	0.506	0.41 (0.59)	0.486
Alcohol		0.01 (0.01)	0.558	0.001 (0.01)	0.952
Physical activity (Cambridge Index)	Moderately inactive	0.27 (0.58)	0.645	-	
	Moderately active	1.26 (0.68)	0.065	-	
	Active	1.12 (0.81)	0.168	-	
Hormone replacement therapy*	Yes	-	-	0.26 (0.59)	0.657

* Since hormone therapy is a women specific variable, gender was no longer included in the model

Supplementary Table 5. Sensitivity analyses using alternative dichotomisation for education by including 'professionals' in the 'low' education class.

**(A) Plasma concentration of CSF3**		**Model A**		**Model B-1**		**Model B-2**		**Model C**		**Fully Adjusted Model**	
**Variables**	**Levels**	β **(SE)**	**P-value**	β **(SE)**	**P-value**	β **(SE)**	**P-value**	β **(SE)**	**P-value**	β **(SE)**	**P-value**
Father's occupational position	Manual	0.29 (0.09)	0.002	0.26 (0.10)	0.011	0.29 (0.10)	0.004	0.26 (0.10)	0.012	0.27 (0.10)	0.010
Participant's education	Low			0.07 (0.11)	0.500	-	-	0.08 (0.12)	0.493	0.06 (0.12)	0.608
Household's highest occupation	Manual					0.01 (0.10)	0.916	-0.02 (0.11)	0.872	-0.03 (0.11)	0.773
BMI										0.02 (0.01)	0.239
Smoking status	Former									-0.01 (0.12)	0.947
	Current									0.06 (0.12)	0.619
Alcohol										0.001 (0.003)	0.654
**(B) Inflammatory score**											
**Variables**	**Levels**	β **(SE)**	**P-value**	β **(SE)**	**P-value**	β **(SE)**	**P-value**	β **(SE)**	**P-value**	β **(SE)**	**P-value**
Father's occupational position	Manual	1.96 (0.89)	0.029	2.36 (0.98)	0.017	2.64 (0.93)	0.005	2.68 (0.99)	0.007	2.54 (1.00)	0.012
Participant's education	Low			-1.02 (1.03)	0.324	-	-	-0.15 (1.11)	0.893	-0.09 (1.13)	0.939
Household's highest occupation	Manual					-2.22 (0.97)	0.023	-2.17 (1.05)	0.041	-2.08 (1.07)	0.053
BMI										-0.09 (0.13)	0.498
Smoking status	Former									-0.56 (1.16)	0.631
	Current									-0.58 (1.17)	0.617
Alcohol										-0.02 (0.03)	0.450
**(C) Principal component 1**											
**Variables**	**Levels**	β **(SE)**	**P-value**	β **(SE)**	**P-value**	β **(SE)**	**P-value**	β **(SE)**	**P-value**	β **(SE)**	**P-value**
Father's occupational position	Manual	-0.60 (0.45)	0.182	-0.86 (0.49)	0.078	-0.84 (0.47)	0.074	-0.96 (0.49)	0.053	-0.92 (0.50)	0.069
Participant's education	Low			0.68 (0.51)	0.184	-	-	0.43 (0.56)	0.442	0.43 (0.56)	0.449
Household's highest occupation	Manual					0.79 (0.49)	0.104	0.64 (0.53)	0.227	0.65 (0.54)	0.227
BMI										0.001 (0.07)	0.984
Smoking status	Former									0.27 (0.58)	0.645
	Current									0.48 (0.58)	0.412
Alcohol										0.01 (0.01)	0.671

Social mobility with participant educational level*

**(A) Inflammatory score**			
**Variables**	β	**SE**	**P-value**
Intercept (Non-Manual – High)	8.04	3.24	0.014
Manual to High	4.12	1.60	0.011
Non Manual to Low	0.34	1.42	0.810
Manual – Low	1.69	1.12	0.132
**(B) Principal component 1**			
**Variables**	β	**SE**	**P-value**
Intercept (Non-Manual – High)	-1.67	1.62	0.303
Manual to High	-1.44	0.80	0.072
Non Manual to Low	0.24	0.71	0.739
Manual – Low	-0.30	0.56	0.598

* Model adjusted on age, gender, NHL case, BC cases, phase, center

Supplementary Table 6. Sensitivity analyses. Results are presented for healthy controls only (N=115)

**(A) Plasma concentration of CSF3**		**Model A***		**Model B-1**		**Model B-2**		**Model C**		**Fully Adjusted Model**	
**Variables**	**Levels**	**β (SE)**	**P-value**	**β (SE)**	**P-value**	**β (SE)**	**P-value**	**β (SE)**	**P-value**	**β (SE)**	**P-value**
Father's occupational position	Manual	0.27 (0.12)	0.030	0.30 (0.14)	0.031	0.28 (0.13)	0.034	0.30 (0.14)	0.032	0.27 (0.14)	0.059
Participant's education	Low			-0.07 (0.14)	0.626	-	-	-0.07 (0.16)	0.647	-0.08 (0.16)	0.620
Household's highest occupation	Manual					-0.02 (0.13)	0.854	0.01 (0.15)	0.940	0.02 (0.15)	0.878
BMI										0.002 (0.017)	0.906
Smoking status	Former									-0.03 (0.17)	0.855
	Current									-0.10 (0.00)	0.563
Alcohol										-0.005 (0.004)	0.227
**(B) Inflammatory score**											
**Variables**	**Levels**	**β (SE)**	**P-value**	**β (SE)**	**P-value**	**β (SE)**	**P-value**	**β (SE)**	**P-value**	**β (SE)**	**P-value**
Father's occupational position	Manual	3.03 (1.31)	0.023	4.23 (1.44)	0.004	3.62 (1.36)	0.009	4.28 (1.45)	0.004	4.34 (1.51)	0.005
Participant's education	Low			-2.71 (1.43)	0.060	-	-	-2.20 (1.67)	0.189	-2.41 (1.72)	0.164
Household's highest occupation	Manual					-1.94 (1.32)	0.143	-0.91 (1.53)	0.551	-0.97 (1.56)	0.534
BMI										0.07 (0.19)	0.723
Smoking status	Former									-0.85 (1.81)	0.640
	Current									-1.00 (1.77)	0.572
Alcohol										0.01 (0.04)	0.861
**(C) Principal component 1**											
**Variables**	**Levels**	**β (SE)**	**P-value**	**β (SE)**	**P-value**	**β (SE)**	**P-value**	**β (SE)**	**P-value**	**β (SE)**	**P-value**
Father's occupational position	Manual	-0.85 (0.65)	0.197	-1.35 (0.72)	0.065	-0.99 (0.68)	0.152	-1.34 (0.73)	0.068	-1.38 (0.75)	0.071
Participant's education	Low			1.13 (0.71)	0.118	-	-	1.17 (0.83)	0.163	1.32 (0.86)	0.129
Household's highest occupation	Manual					0.47 (0.66)	0.482	-0.08 (0.77)	0.914	-0.06 (0.78)	0.937
BMI										-0.07 (0.09)	0.467
Smoking status	Former									0.38 (0.90)	0.674
	Current									0.57 (0.88)	0.517
Alcohol										0.001 (0.02)	0.962

Social mobility with highest household occupational position*

**(A) Inflammatory score**			
**Variables**	**β**	**SE**	**P-value**
Intercept (stable Non-manual)	2.89	4.57	0.528
Manual to Non-manual	2.98	1.60	0.065
Non-Manual to Manual	-3.81	2.75	0.168
stable Manual	1.55	1.61	0.338
**(B) Principal component 1**			
**Variables**	**β**	**SE**	**P-value**
Intercept (stable Non-manual)	1.54	2.29	0.502
Manual to Non-manual	-0.71	0.80	0.378
Non-Manual to Manual	1.28	1.38	0.356
stable Manual	-0.47	0.81	0.563

* Model adjusted on age, gender, phase, disease and center

Supplementary Table 7. Summary features of the 28 inflammatory markers included in the study.

Manufacturer's name	LOD (pg/mL)	Protein	Official Symbol*	Entrez ID*	Cellular components*	Biological processes*	Molecular function*
eotaxin	1.20	Chemokine (C-C motif) ligand 11	CCL11	6356	Cytoplasm; Extracellular	Cell communication ; Signal transduction	Chemokine activity
mcp-1	0.73	Monocyte chemotactic protein 1	CCL2	6347	Extracellular	Immune response	Chemokine activity
mcp-3	1.79	chemokine (C-C motif) ligand 7	CCL7	6354	Extracellular	Cell communication ; Signal transduction	Chemokine activity
mip-1a	0.45	chemokine (C-C motif) ligand 3	CCL3	6348	Cytoplsam; Extracellular	Cell communication ; Signal transduction	Chemokine activity
mip-1b	1.75	chemokine (C-C motif) ligand 4	CCL4	6351	Extracellular	Cell communication ; Signal transduction	Chemokine activity
mdc	2.25	chemokine (C-C motif) ligand 22	CCL22	6367	Extracellular	Cell communication ; Signal transduction	Chemokine activity
fractalkine	1.30	chemokine (C-X3-C motif) ligand 1	CX3CL1	6376	Extracellular; plasma menbrane	Cell communication ; Signal transduction	Chemokine activity
gro	3.80	chemokine (C-X-C motif) ligand 1	CXCL1	2919	Extracellular	Immune response	Chemokine activity
ip-10	2.81	CXCL10 chemokine (C-X-C motif) ligand 10	CXCL10	3627	Extracellular	Immune response	Chemokine activity
il-8	0.07	Interleukin 8 //chemokine (C-X-C motif) ligand 8	IL8 // CXCL8	3576	Extracellular	Immune response	Chemokine activity
infa 2	1.54	interferon, alpha 2	IFNA2	3440	Extracellular	Immune response	Cytokine activity
il-1b	0.08	interleukin 1, beta	IL1B	3553	Cytoplasm; Extracellular; nucleus; plasma membrane	Immune response	Cytokine activity
il-2	0.09	interleukin 2	IL2	3558	Extracellular	Immune response	Cytokine activity
il-4	0.11	interleukin 4	IL4	3565	Extracellular	Immune response	Cytokine activity
il-5	0.04	interleukin 5	IL5	3567	Extracellular	Immune response	Cytokine activity
il-6	0.02	interleukin 6	IL6	3569	Extracellular	Immune response	Cytokine activity
il-7	0.08	interleukin 7	IL7	3574	Extracellular	Immune response	Cytokine activity
il-10	0.10	interleukin 10	IL10	3586	Extracellular	Immune response	Cytokine activity
il-13	0.04	interleukin 13	IL13	3596	Extracellular	Immune response	Cytokine activity
infg	0.14	interferon, gamma	IFNG	3458	Extracellular; nucleus	Immune response	Cytokine activity
scd40l		soluble CD40 ligand	CD40LG	959	Extracellular; plasma menbrane	Immune response	Cytokine activity
tnfa	0.01	tumor necrosis factor	TNF	7124	Extracellular; plasma menbrane	Cell communication; Signal transduction	Cytokine activity / receptor binding
egf	1.73	epidermal growth factor	EGF	1950	Nucleus; plasma membrane	Cell communication; Signal transduction	Growth factor activity
g-csf	0.71	colony stimulating factor 3 (granulocyte)	CSF3	1440	Extracellular	Immune response	growth factor activity
gm-csf	0.08	colony stimulating factor 2 (granulocyte-macrophage)	CSF2	1437	Extracellular	Immune response	growth factor activity
tgfa	0.28	transforming growth factor, alpha	TGFA	7039	Cytoplasm; endoplasmic reticulum; nucleus; plasma membrane	Cell communication ; Signal transduction	Growth factor activity
vegf	8.83	vascular endothelial growth factor A	VEGFA	7422	Extracellular	Cell communication ; Signal transduction	Growth factor activity
fgf2	2.21	fibroblast growth factor 2	FGF2	2247	Extracellular; nucleus	Cell communication; Signal transduction	Mitogenic and angiogenic activities

* plasma proteome database and pubmed annotation

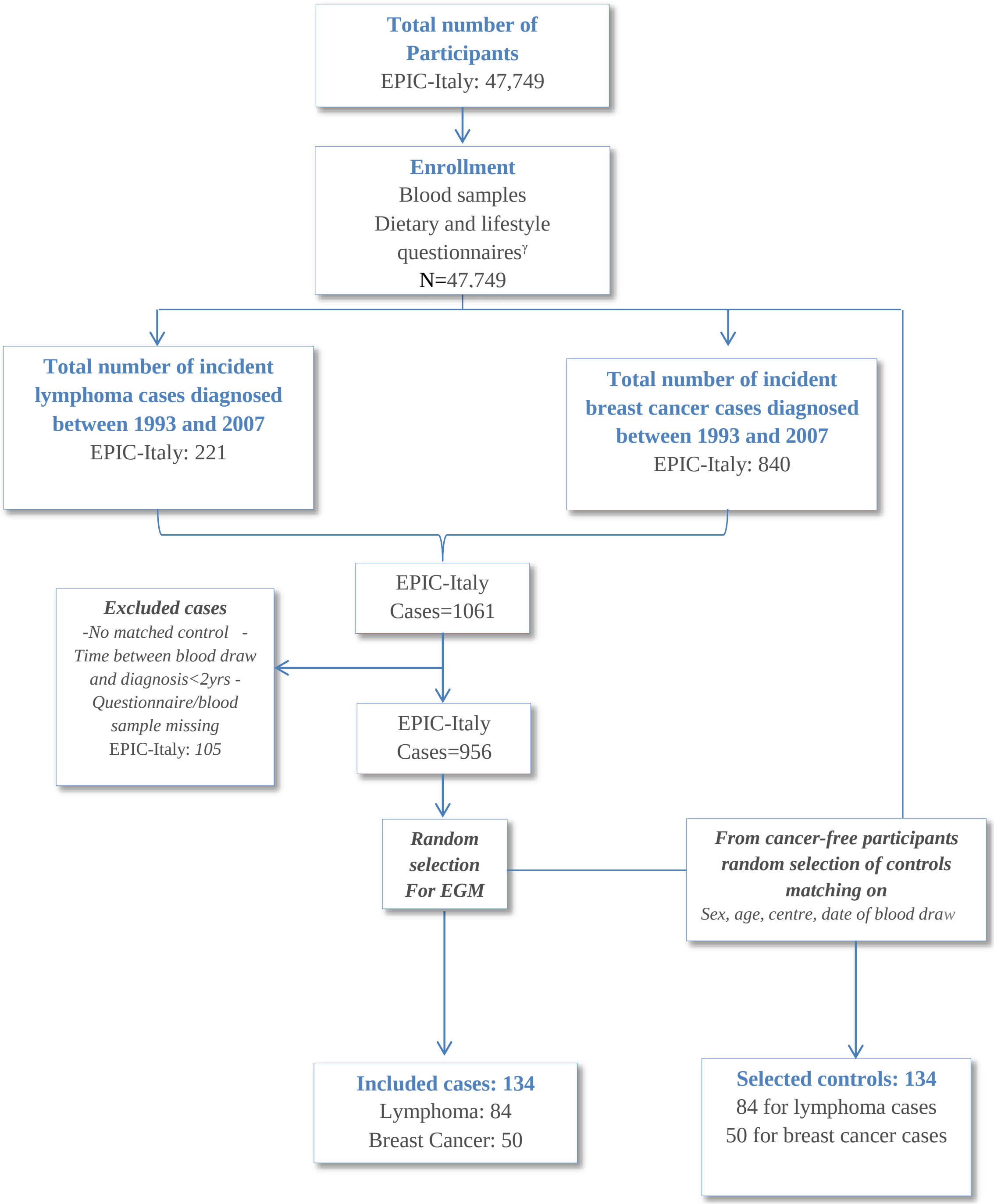

γ: occupational questionnaire is missing for one EPIC-Italy centre (Napoli)

Supplementary Figure 1. Flow chart summarising the participants selection procedure.

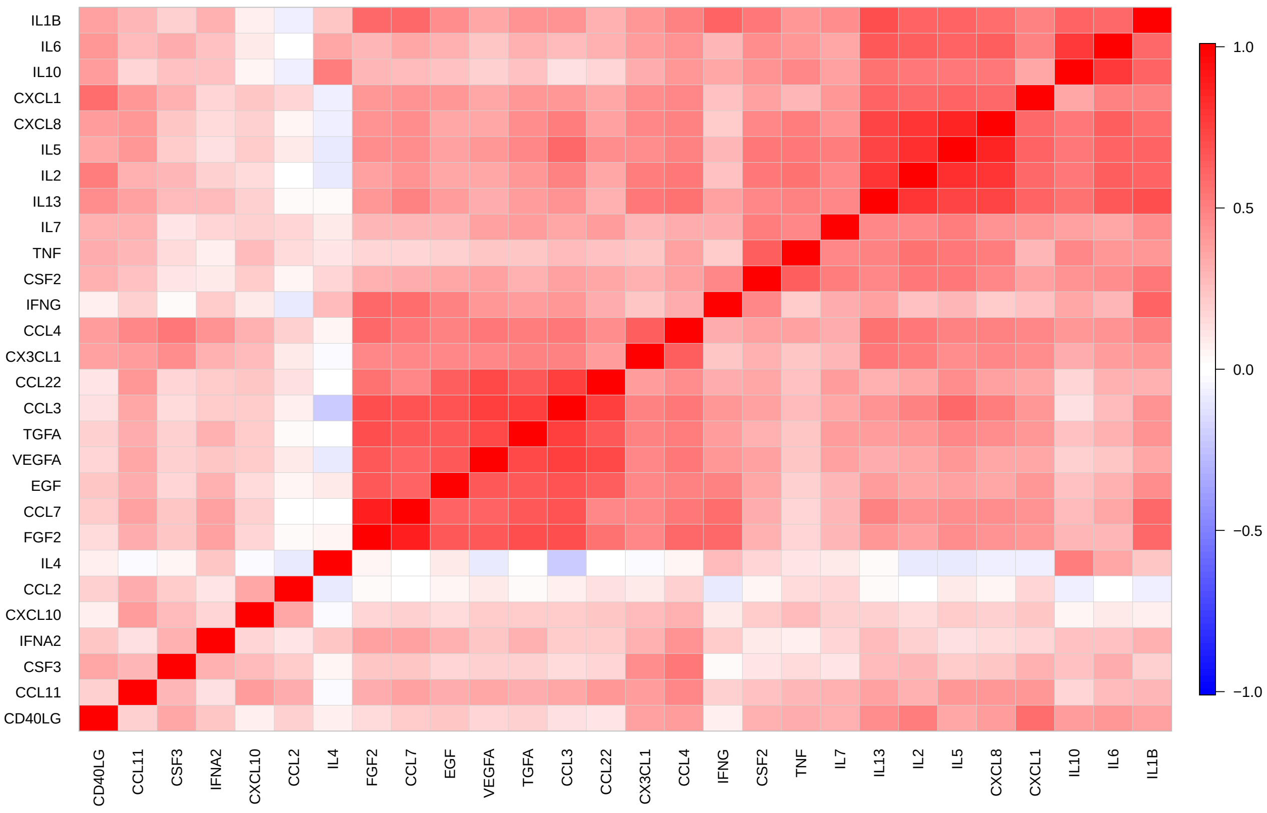

Supplementary Figure 2. Heatmap representing the pairwise Spearman correlation for all 28 proteins assayed in the (N=268) Epic-Italy participants. Proteins abbreviations and functional features are described in Supplementary Table S7.

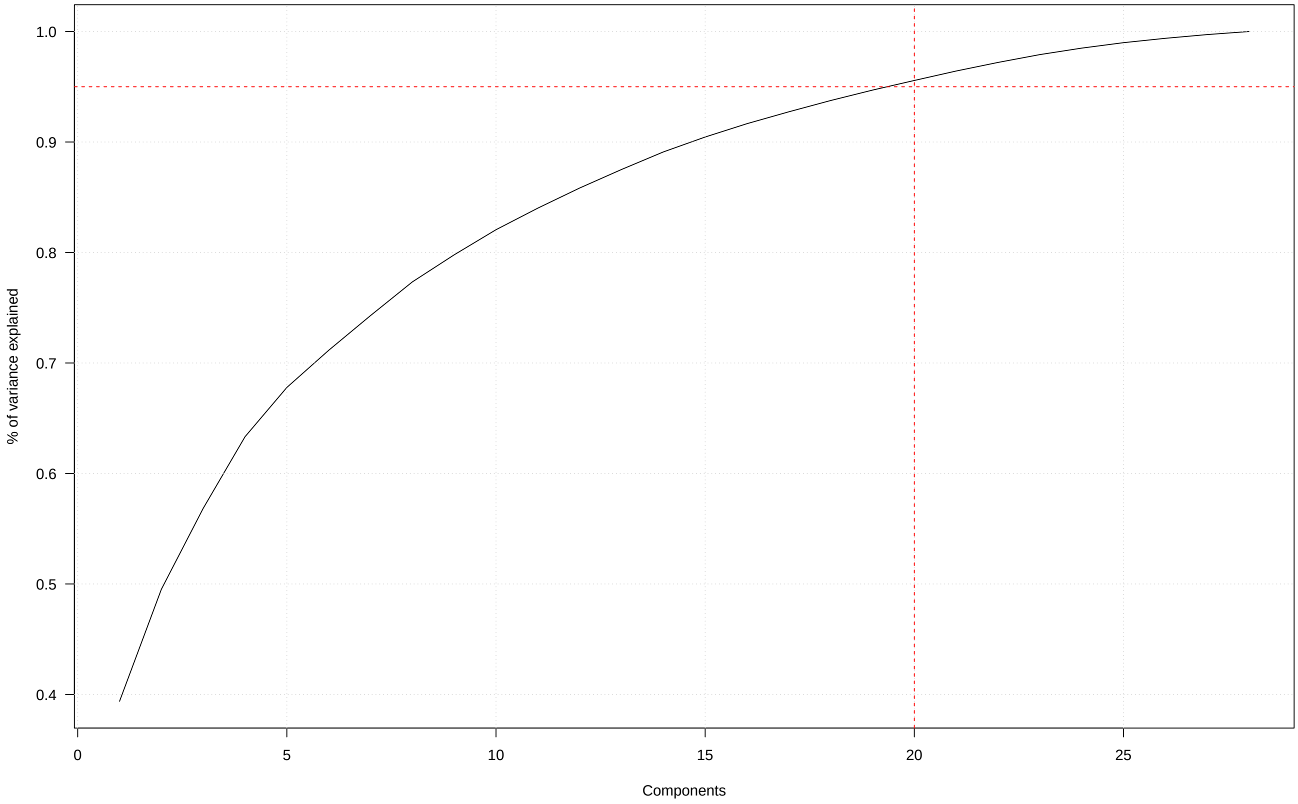

Supplementary Figure 3. Scree plot from the PCA of the 28 inflammatory proteins levels.
